# Supplementary material for: A Pair of Prognostic Biomarkers in Triple-Negative Breast Cancer: KLK10 and KLK11 mRNA Expression
Source: Life (Basel). 2022 Sep 28;12(10):1517. doi: 10.3390/life12101517 (PMC9605449; doi:10.3390/life12101517)
Supplement: Supplementary file 1 [file life-12-01517-s001.zip › life-1909714-supplementary.pdf]

A

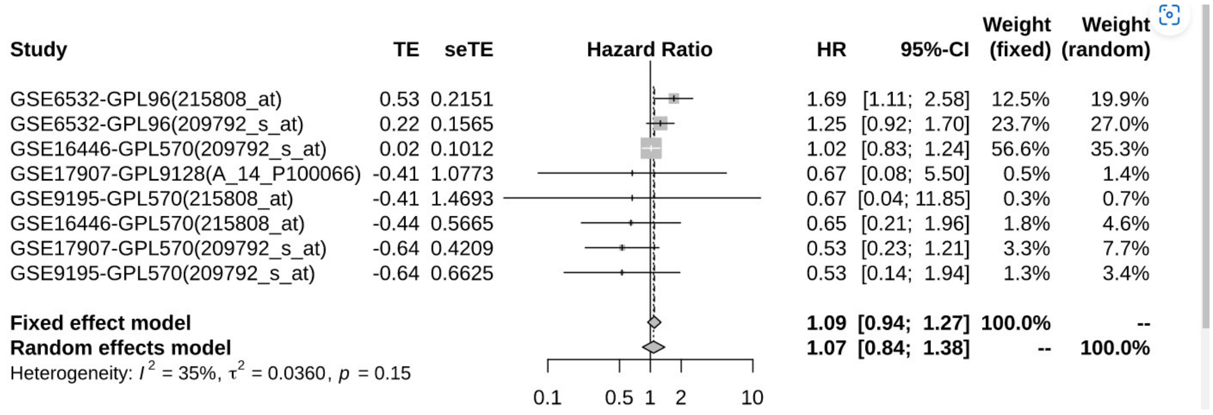

B

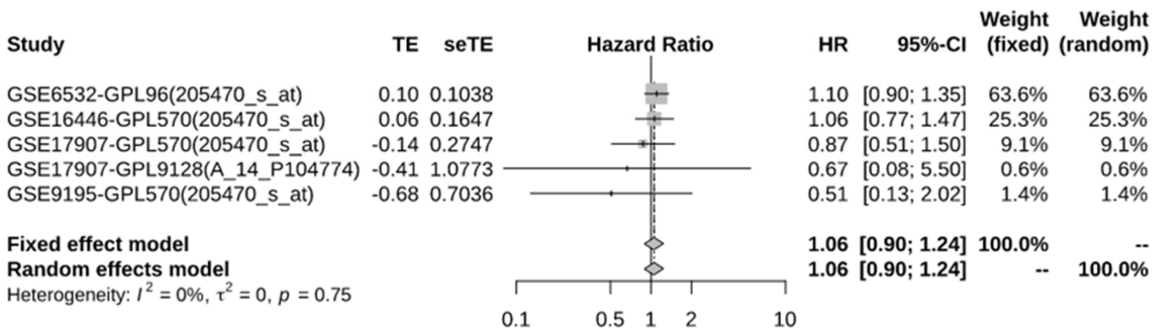

Figure S1. Forest plot of the impact of KLK10 (A) and KLK11 (B) expression on the hazard ratio of progression-free survival in breast cancer. Depicted are several publicly available datasets. The analysis was performed via the GENT2 platform.

Study	TE	seTE	HR	95%-CI	Weight (fixed)	Weight (random)
GSE31589-GPL4133(6020)	2.05	2.6146	7.75	[0.05; 1302.09]	0.0%	0.0%
GSE31589-GPL4133(23803)	0.78	1.3342	2.19	[0.16; 29.86]	0.0%	0.0%
GSE31589-GPL8264(237189)	0.70	1.2486	2.02	[0.17; 23.35]	0.0%	0.0%
GSE20711-GPL570(215808_at)	0.69	0.9235	1.99	[0.33; 12.17]	0.0%	0.0%
GSE20713-GPL570(215808_at)	0.69	0.9235	1.99	[0.33; 12.17]	0.0%	0.0%
GSE31448-GPL570(215808_at)	0.40	0.2774	1.49	[0.86; 2.56]	0.0%	0.0%
GSE21653-GPL570(215808_at)	0.34	0.2800	1.40	[0.81; 2.42]	0.0%	0.0%
GSE25055-GPL96(215808_at)	0.26	0.1797	1.29	[0.91; 1.84]	0.0%	0.0%
GSE22210-GPL9183(KLK10_P268_R)	0.26	0.5429	1.29	[0.45; 3.74]	0.0%	0.0%
GSE25065-GPL96(215808_at)	0.23	0.1919	1.26	[0.87; 1.84]	0.0%	0.0%
GSE2603-GPL96(215808_at)	0.18	0.2814	1.20	[0.69; 2.09]	0.0%	0.0%
GSE7390-GPL96(209792_s_at)	0.13	0.1170	1.14	[0.91; 1.43]	0.0%	0.0%
GSE25055-GPL96(209792_s_at)	0.09	0.0629	1.10	[0.97; 1.24]	0.0%	0.0%
GSE25065-GPL96(209792_s_at)	0.09	0.0772	1.10	[0.94; 1.28]	0.0%	0.0%
GSE4922-GPL96(209792_s_at)	0.09	0.0871	1.10	[0.93; 1.30]	0.0%	0.0%
GSE15363-GPL1708(26344)	0.08	0.0991	1.09	[0.89; 1.32]	0.0%	0.0%
GSE1456-GPL96(209792_s_at)	0.08	0.2323	1.08	[0.69; 1.71]	0.0%	0.0%
GSE12093-GPL96(215808_at)	0.06	0.1736	1.06	[0.76; 1.50]	0.0%	0.0%
GSE58984-GPL570(215808_at)	0.04	0.0261	1.04	[0.99; 1.10]	0.0%	0.1%
GSE31589-GPL8264(212843)	0.04	1.0756	1.04	[0.13; 8.58]	0.0%	0.0%
GSE48391-GPL570(215808_at)	0.03	0.0125	1.03	[1.00; 1.05]	0.1%	0.4%
GSE31448-GPL570(209792_s_at)	0.01	0.0623	1.01	[0.90; 1.14]	0.0%	0.0%
GSE42568-GPL570(215808_at)	0.00	0.0037	1.00	[1.00; 1.01]	0.6%	4.1%
GSE21653-GPL570(209792_s_at)	0.00	0.0627	1.00	[0.89; 1.13]	0.0%	0.0%
GSE42568-GPL570(209792_s_at)	0.00	0.0006	1.00	[1.00; 1.00]	24.9%	20.8%
GSE58984-GPL570(209792_s_at)	0.00	0.0005	1.00	[1.00; 1.00]	29.8%	21.1%
GSE88770-GPL570(209792_s_at)	0.00	0.0006	1.00	[1.00; 1.00]	20.8%	20.4%
GSE103091-GPL570(209792_s_at)	-0.00	0.0006	1.00	[1.00; 1.00]	21.4%	20.5%
GSE103091-GPL570(215808_at)	-0.00	0.0073	1.00	[0.99; 1.01]	0.1%	1.2%
GSE48391-GPL570(209792_s_at)	-0.00	0.0019	1.00	[1.00; 1.00]	2.3%	10.8%
GSE31589-GPL8264(89054)	-0.01	1.2472	0.99	[0.09; 11.40]	0.0%	0.0%
GSE16446-GPL570(209792_s_at)	-0.02	0.1332	0.99	[0.76; 1.28]	0.0%	0.0%
GSE20685-GPL570(209792_s_at)	-0.02	0.0480	0.98	[0.89; 1.08]	0.0%	0.0%
GSE88770-GPL570(215808_at)	-0.02	0.0161	0.98	[0.95; 1.01]	0.0%	0.3%
GSE2603-GPL96(209792_s_at)	-0.03	0.0903	0.97	[0.81; 1.16]	0.0%	0.0%
GSE31589-GPL8269(168302)	-0.08	0.1813	0.93	[0.65; 1.32]	0.0%	0.0%
GSE20685-GPL570(215808_at)	-0.08	0.0790	0.92	[0.79; 1.08]	0.0%	0.0%
GSE31589-GPL8269(169600)	-0.08	0.1786	0.92	[0.65; 1.31]	0.0%	0.0%
GSE31589-GPL8269(152052)	-0.11	0.2004	0.90	[0.61; 1.33]	0.0%	0.0%
GSE31589-GPL8269(132717)	-0.11	0.2375	0.90	[0.56; 1.43]	0.0%	0.0%
GSE31589-GPL8269(194547)	-0.13	0.1741	0.88	[0.63; 1.24]	0.0%	0.0%
GSE31589-GPL8269(45114)	-0.17	0.2358	0.85	[0.53; 1.34]	0.0%	0.0%
GSE31589-GPL8264(20166)	-0.17	1.4703	0.84	[0.05; 15.04]	0.0%	0.0%
GSE12093-GPL96(209792_s_at)	-0.17	0.1511	0.84	[0.63; 1.13]	0.0%	0.0%
GSE7390-GPL96(215808_at)	-0.17	0.7960	0.84	[0.18; 4.00]	0.0%	0.0%
GSE33926-GPL7264(A_23_P107911)	-0.27	0.1914	0.76	[0.52; 1.11]	0.0%	0.0%
GSE15363-GPL1708(6662)	-0.29	0.5855	0.75	[0.24; 2.37]	0.0%	0.0%
GSE20711-GPL570(209792_s_at)	-0.29	0.1796	0.75	[0.53; 1.06]	0.0%	0.0%
GSE20713-GPL570(209792_s_at)	-0.29	0.1796	0.75	[0.53; 1.06]	0.0%	0.0%
GSE31589-GPL8264(198864)	-0.30	0.7146	0.74	[0.18; 2.99]	0.0%	0.0%
GSE31589-GPL8264(123011)	-0.34	0.6759	0.71	[0.19; 2.68]	0.0%	0.0%
GSE31589-GPL8264(165810)	-0.35	0.6766	0.70	[0.19; 2.65]	0.0%	0.0%
GSE31589-GPL8274(10514)	-0.37	0.2654	0.69	[0.41; 1.16]	0.0%	0.0%
GSE31589-GPL8264(109079)	-0.37	0.9351	0.69	[0.11; 4.31]	0.0%	0.0%
GSE1456-GPL96(215808_at)	-0.39	0.7291	0.67	[0.16; 2.82]	0.0%	0.0%
GSE16446-GPL570(215808_at)	-0.39	0.7146	0.67	[0.17; 2.73]	0.0%	0.0%
GSE31589-GPL8264(100553)	-0.40	1.1745	0.67	[0.07; 6.72]	0.0%	0.0%
GSE31589-GPL8274(21150)	-0.41	0.2694	0.67	[0.39; 1.13]	0.0%	0.0%
GSE31589-GPL8274(25196)	-0.41	0.2674	0.66	[0.39; 1.12]	0.0%	0.0%
GSE31589-GPL8264(150229)	-0.42	0.9694	0.66	[0.10; 4.41]	0.0%	0.0%
GSE31589-GPL8264(58339)	-0.42	0.6978	0.65	[0.17; 2.57]	0.0%	0.0%
GSE31589-GPL8274(76004)	-0.46	0.3062	0.63	[0.35; 1.15]	0.0%	0.0%
GSE31589-GPL8274(104833)	-0.46	0.2970	0.63	[0.35; 1.13]	0.0%	0.0%
GSE31589-GPL8264(71570)	-0.49	0.6846	0.61	[0.16; 2.34]	0.0%	0.0%
GSE31589-GPL8269(73825)	-0.52	0.5356	0.60	[0.21; 1.70]	0.0%	0.0%
GSE31589-GPL8264(112846)	-0.54	0.9111	0.58	[0.10; 3.47]	0.0%	0.0%
GSE4922-GPL96(215808_at)	-0.66	0.3711	0.51	[0.25; 1.07]	0.0%	0.0%
GSE31589-GPL8269(217731)	-0.87	0.6528	0.42	[0.12; 1.51]	0.0%	0.0%
GSE31589-GPL8274(136595)	-0.88	0.4037	0.41	[0.19; 0.91]	0.0%	0.0%
GSE31589-GPL8269(139673)	-0.94	0.6745	0.39	[0.10; 1.47]	0.0%	0.0%
GSE31589-GPL8274(134939)	-1.01	0.4977	0.36	[0.14; 0.97]	0.0%	0.0%
GSE31589-GPL8274(228094)	-1.05	0.4782	0.35	[0.14; 0.90]	0.0%	0.0%
GSE31589-GPL8274(131751)	-1.08	0.4855	0.34	[0.13; 0.88]	0.0%	0.0%
GSE31589-GPL8274(81507)	-1.10	0.5643	0.33	[0.11; 1.01]	0.0%	0.0%
GSE31589-GPL8274(93358)	-1.11	0.5724	0.33	[0.11; 1.01]	0.0%	0.0%
GSE31589-GPL8269(89267)	-1.14	0.6704	0.32	[0.09; 1.19]	0.0%	0.0%
GSE31589-GPL8274(127862)	-1.28	0.5451	0.28	[0.10; 0.81]	0.0%	0.0%
GSE31589-GPL8274(204539)	-1.41	0.6255	0.24	[0.07; 0.83]	0.0%	0.0%
**Fixed effect model**			**1.00**	**[1.00; 1.00]**	**100.0%**	**--**
**Random effects model**			**1.00**	**[1.00; 1.00]**	**--**	**100.0%**

Heterogeneity: $I^2 = 28\%$, $\tau^2 < 0.0001$, $p = 0.01$

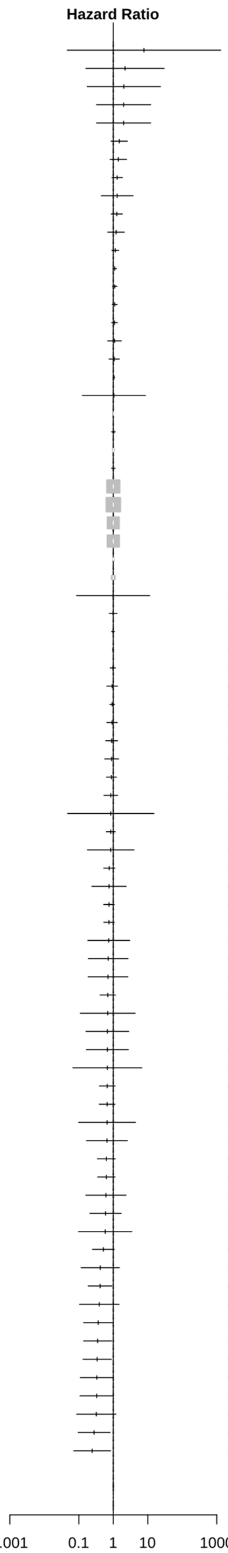

Figure S2. Forest plot of the impact of KLK10 expression on the hazard ratio of overall survival in breast cancer. Depicted are several publicly available datasets. The analysis was performed via the GENT2 platform.

Study	TE	seTE	HR	95%-CI	Weight (fixed)	Weight (random)
GSE4922-GPL96(205470_s_at)	0.05	0.0789	1.05	[0.90; 1.23]	0.0%	0.0%
GSE33926-GPL7264(A_23_P101500)	0.02	0.2739	1.02	[0.60; 1.75]	0.0%	0.0%
GSE31448-GPL570(205470_s_at)	0.00	0.0842	1.00	[0.85; 1.18]	0.0%	0.0%
GSE58984-GPL570(205470_s_at)	0.00	0.0010	1.00	[1.00; 1.00]	2.4%	13.3%
GSE88770-GPL570(205470_s_at)	-0.00	0.0002	1.00	[1.00; 1.00]	66.8%	26.3%
GSE42568-GPL570(205470_s_at)	-0.00	0.0003	1.00	[1.00; 1.00]	20.6%	24.3%
GSE48391-GPL570(205470_s_at)	-0.00	0.0007	1.00	[1.00; 1.00]	5.8%	19.0%
GSE103091-GPL570(205470_s_at)	-0.00	0.0008	1.00	[1.00; 1.00]	4.3%	17.2%
GSE16446-GPL570(205470_s_at)	-0.00	0.2367	1.00	[0.63; 1.59]	0.0%	0.0%
GSE21653-GPL570(205470_s_at)	-0.01	0.0850	0.99	[0.84; 1.17]	0.0%	0.0%
GSE12093-GPL96(205470_s_at)	-0.03	0.0977	0.97	[0.80; 1.18]	0.0%	0.0%
GSE7390-GPL96(205470_s_at)	-0.03	0.1219	0.97	[0.76; 1.23]	0.0%	0.0%
GSE20685-GPL570(205470_s_at)	-0.06	0.0615	0.94	[0.84; 1.06]	0.0%	0.0%
GSE25065-GPL96(205470_s_at)	-0.09	0.0927	0.91	[0.76; 1.09]	0.0%	0.0%
GSE2603-GPL96(205470_s_at)	-0.10	0.1154	0.90	[0.72; 1.13]	0.0%	0.0%
GSE25055-GPL96(205470_s_at)	-0.11	0.0783	0.89	[0.77; 1.04]	0.0%	0.0%
GSE1456-GPL96(205470_s_at)	-0.16	0.1869	0.85	[0.59; 1.23]	0.0%	0.0%
GSE22210-GPL9183(KLK11_P103_R)	-0.17	0.5654	0.84	[0.28; 2.55]	0.0%	0.0%
GSE20713-GPL570(205470_s_at)	-0.19	0.1688	0.83	[0.60; 1.16]	0.0%	0.0%
GSE20711-GPL570(205470_s_at)	-0.19	0.1688	0.83	[0.60; 1.16]	0.0%	0.0%
GSE15363-GPL1708(16748)	-0.19	0.2531	0.83	[0.50; 1.36]	0.0%	0.0%
GSE31589-GPL8274(73909)	-0.36	0.3544	0.70	[0.35; 1.40]	0.0%	0.0%
GSE31589-GPL8274(61428)	-0.41	0.3706	0.67	[0.32; 1.38]	0.0%	0.0%
GSE31589-GPL8274(69519)	-0.51	0.3706	0.60	[0.29; 1.24]	0.0%	0.0%
GSE31589-GPL8274(10727)	-0.54	0.2721	0.58	[0.34; 0.99]	0.0%	0.0%
GSE31589-GPL8274(147826)	-0.57	0.2636	0.57	[0.34; 0.95]	0.0%	0.0%
GSE31589-GPL8274(152245)	-0.60	0.2930	0.55	[0.31; 0.97]	0.0%	0.0%
GSE31589-GPL8269(158188)	-0.60	0.4319	0.55	[0.23; 1.28]	0.0%	0.0%
GSE31589-GPL8269(96520)	-0.62	0.3890	0.54	[0.25; 1.15]	0.0%	0.0%
GSE31589-GPL8264(75703)	-0.63	0.7229	0.54	[0.13; 2.21]	0.0%	0.0%
GSE31589-GPL8274(205035)	-0.65	0.3232	0.52	[0.28; 0.99]	0.0%	0.0%
GSE31589-GPL8264(242078)	-0.67	0.8793	0.51	[0.09; 2.87]	0.0%	0.0%
GSE31589-GPL8269(109408)	-0.67	0.3805	0.51	[0.24; 1.07]	0.0%	0.0%
GSE31589-GPL8264(103518)	-0.68	0.7366	0.51	[0.12; 2.15]	0.0%	0.0%
GSE31589-GPL8274(229079)	-0.70	0.3270	0.50	[0.26; 0.94]	0.0%	0.0%
GSE31589-GPL8264(136213)	-0.71	0.7731	0.49	[0.11; 2.24]	0.0%	0.0%
GSE31589-GPL8269(80193)	-0.72	0.4055	0.49	[0.22; 1.08]	0.0%	0.0%
GSE31589-GPL8264(92683)	-0.74	0.8794	0.48	[0.08; 2.67]	0.0%	0.0%
GSE31589-GPL8269(148819)	-0.87	0.3975	0.42	[0.19; 0.91]	0.0%	0.0%
GSE31589-GPL8264(162479)	-0.89	1.0323	0.41	[0.05; 3.12]	0.0%	0.0%
GSE31589-GPL8269(35364)	-0.93	0.4265	0.39	[0.17; 0.91]	0.0%	0.0%
GSE31589-GPL8264(184181)	-1.06	1.1167	0.35	[0.04; 3.09]	0.0%	0.0%
GSE31589-GPL8264(164579)	-1.10	1.1418	0.33	[0.04; 3.11]	0.0%	0.0%
GSE22210-GPL9183(KLK11_P1290_F)	-1.77	1.5229	0.17	[0.01; 3.38]	0.0%	0.0%
**Fixed effect model**			**1.00**	**[1.00; 1.00]**	**100.0%**	**--**
**Random effects model**			**1.00**	**[1.00; 1.00]**	**--**	**100.0%**

Heterogeneity: $I^2 = 31\%$, $\tau^2 < 0.0001$, $p = 0.03$

Figure S3. Forest plot of the impact of KLK11 expression on the hazard ratio of overall survival in breast cancer. Depicted are several publicly available datasets. The analysis was performed via the GENT2 platform.

Table S1. Multivariate Cox regression analysis of clinical patient outcome in triple-negative breast cancer for clinicopathological parameters (including chemotherapy) and tumor biological factors.

Clinicopathological parameters	DFS			OS		
	No[a]	HR (95% CI)[b]	p	No[a]	HR (95% CI)[b]	p
**Age**			0.186			**0.030**
≤ 60 years	57	1		57	1	
> 60 years	41	1.61 (0.80-3.24)		43	2.36 (1.09-5.112)	
**Lymph node status**			0.118			0.245
N0	53	1		55	1	
N^+	45	1.71 (0.87-3.34)		45	1.51 (0.75-3.04)	
**Tumor Size**			0.419			0.590
≤20 mm	28	1		29	1	
>20 mm	70	1.39 (0.63-3.06)		71	1.27 (0.54-2.98)	
**Chemotherapy**			**0.003**			**0.016**
no	19	1		20	1	
yes	79	0.31 (0.144-0.66)		80	0.39 (0.18-0.84)	
**KLK10 mRNA**[c]			**0.045**			0.147
low	64	1		66	1	
high	34	1.94 (1.01-3.71)		34	1.69 (0.83-3.43)	
**KLK11 mRNA**[c]			0.253			0.108
low	51	1		53	1	
high	47	1.47 (0.76-2.87)		47	1.81 (0.88-3.74)	

Chi-square test, significant p-values ($p < 0.05$) are indicated in bold.

[a] Number of patients.

[b] HR: hazard ratio (CI: confidence interval) of univariate Cox regression analysis.

[c] dichotomized into low and high levels by the 50th percentile for KLK11 and the 67th percentile for KLK10.

[d] dichotomized into low levels by KLK10 low and KLK11 low and high levels by KLK10 high and/or KLK11 high.
